# Relationship Between Neck Circumference, Central and Overall Obesity Indices, and the Severity of Coronary Artery Disease in Patients With Type 2 Diabetes Mellitus

**DOI:** 10.7759/cureus.87136

**Published:** 2025-07-01

**Authors:** Nagwa A Lachine, Magdy H Megallaa, Mohamed A Sadaka, Mona M Tahoun, Dalia H Assad, Amr Y Elfeky, Mohammed H Zeitoun

**Affiliations:** 1 Department of Diabetes and Metabolism, Alexandria University, Alexandria, EGY; 2 Department of Cardiology and Angiology, Alexandria University, Alexandria, EGY; 3 Department of Clinical and Chemical Pathology, Alexandria University, Alexandria, EGY; 4 Department of Internal Medicine, Abou Qir General Hospital, Alexandria, EGY

**Keywords:** anthropometric measurements, coronary artery disease, gensini score, neck circumference, type 2 diabetes

## Abstract

Introduction: Coronary artery disease (CAD) is a public health problem and a major morbidity and mortality cause in patients with diabetes. Obesity is a well-established, independent, and modifiable risk factor for CAD that also augments other CAD risk factors, such as hypertension, dyslipidemia, and diabetes mellitus. Although body mass index (BMI) is the cornerstone of the obesity classification system, it does not provide information on fat distribution, which is a crucial element in the relationship between obesity and several metabolic disorders. Waist circumference (WC) and waist-to-hip ratio (WHR) are more reliable anthropometric indicators of visceral adipose tissue that more accurately anticipate obesity-related cardiovascular risks. Neck circumference (NC) is a promising tool for evaluating obesity, and there is growing evidence that increased NC is linked to various cardiovascular diseases. The present study was carried out to evaluate the association between NC and other anthropometric measurements with the severity and extension of CAD in Egyptian patients with type 2 diabetes mellitus (T2DM).

Methods: This case-control study was conducted on 160 T2DM patients recruited from the catheter lab of Alexandria Main University Hospital, Egypt. The participants were divided into two groups: 80 patients with angiographically proven hemodynamically significant CAD (cases) and 80 patients without hemodynamically significant CAD (controls). Obesity was assessed by body weight, BMI, WC, WHR, and NC. The Gensini score (GS) was used to evaluate the severity of CAD based on coronary angiography findings.

Results: CAD patients had significantly higher body weight (adjusted p = 0.049), BMI (adjusted p = 0.0217), WC (adjusted p = 0.0025), WHR (adjusted p = 0.026), and NC (adjusted p = 0.0025). NC was significantly positively correlated with other anthropometric parameters, and it also correlated significantly positively with glycemic parameters such as glycated hemoglobin (adjusted p = 0.00028). Univariate and multivariate analyses of factors influencing the GS revealed, after adjusting for confounding variables, that NC was the only anthropometric measure that remained significantly associated with the GS (p<0.001).

Conclusion: NC is a promising, simple, cost-effective anthropometric measure that can be applied in obesity screening programs. Its strong correlation with CAD severity suggests potential utility as a complementary risk assessment tool. Given its ease of measurement, NC may be considered for incorporation into routine clinical practice.

## Introduction

Coronary artery disease (CAD) remains a significant public health issue and is a leading cause of morbidity and mortality among people with diabetes [1]. Obesity is a well-recognized, independent, and modifiable risk factor for CAD and contributes to the development of other associated risk factors, including hypertension, dyslipidemia, and diabetes mellitus [2]. The regional distribution of body fat is a crucial element in the relationship between obesity and several metabolic disorders. Visceral adipose tissue (VAT) seems to be the most metabolically active adipose tissue depot, which is linked to endothelial dysfunction and subsequent cardiovascular events [3]. However, recent studies have proven that other fat depots, such as upper body subcutaneous adipose tissue (SAT), may also contribute to the development of CAD and may confer risks comparable to VAT [4]. The upper body SAT is the producer of the majority of systemic free fatty acids, causing increased very low-density lipoprotein levels, insulin resistance, and endothelial dysfunction [5]. Studies have proved that upper body SAT increases the risk of metabolic syndrome and is also associated with obstructive sleep apnea syndrome, with repeated hypoxia resulting in endothelial dysfunction and an increase in cardiovascular risk factors [6].

While body mass index (BMI) is widely used to classify obesity, it does not reflect fat distribution, a key factor linking obesity to various metabolic disorders. In contrast, waist circumference (WC) and waist-to-hip ratio (WHR) are more accurate anthropometric measures of visceral fat and better predictors of cardiovascular risk related to obesity [7]. Nevertheless, those measurements can be misleading as they are affected by different causes of abdominal distension and are inconvenient in special populations such as morbidly obese and bedridden people.

Neck circumference (NC) is a simple, reliable, easy-to-measure, and widely available anthropometric measure that acts as a surrogate measure of upper body SAT. It has also been identified as a strong indicator of abdominal obesity and VAT [4]. Emerging evidence suggests that increased NC is associated with multiple cardiovascular risk factors [8]. Given its practicality and the absence of limitations commonly associated with traditional anthropometric measures, NC is increasingly recognized as a valuable tool for assessing obesity and its related cardiometabolic risks.

The Gensini score (GS) is an efficient angiographic scoring system that is used to assess the complexity of CAD and gives beneficial data about the severity and prognosis of CAD. It defines a severity score for each coronary stenosis depending on the grade of luminal narrowing and its geographic importance [9,10].

This study aims to evaluate NC as an independent predictor of CAD severity and to compare its predictive value with that of other traditional anthropometric indices. This study focused on Egyptian patients due to the ease of access and the scarcity of region-specific data regarding the relationship between obesity measures and CAD risk. To the best of our knowledge, it is the first study of the association between NC and CAD diagnosed by coronary angiography in Egyptian patients with type 2 diabetes mellitus (T2DM).

## Materials and methods

Study design

The study was conducted at Alexandria Main University Hospital, Alexandria, Egypt. This study included a case-control comparison between patients with and without angiographically confirmed CAD to assess differences in cardiovascular and anthropometric variables. Additionally, within the CAD group, a cross-sectional analysis was conducted to evaluate the association between GS and various studied factors. A total of 160 T2DM patients were included, comprising 80 patients with angiographically proven hemodynamically significant CAD and 80 patients without hemodynamically significant CAD who constituted the control group. Cases and controls were frequency-matched by age, sex, duration of diabetes, and socioeconomic status to ensure comparability between groups. The sample size was estimated using the Power Analysis and Sample Size (PASS) Version 20 (NCSS, LLC, Utah, USA). Based on a recently published review article, the National Hypertension Project found that the adjusted overall prevalence of CAD in Egypt is estimated to be 8.3% [11]. Thus, the minimal hypothesized sample size of 80 eligible patients (40 per group) is needed, taking into consideration a 95% confidence level and 80% power using the chi-square test. Patients were recruited from the catheter lab of Alexandria Main University Hospital. The study duration was from 1st May 2023 till 28th February 2025. Patients with previous revascularization, advanced chronic hepatic or renal diseases, thyroid disorders or goiter, acute or chronic inflammatory diseases, a history of major pelvic or neck surgery, ascites, any anatomical abnormality or mass lesion of the waist, hip, or neck regions, and pregnant or lactating females were excluded. Although smoking status was recorded, its distribution was similar between cases and controls, minimizing its potential confounding effect on the association between CAD and the other studied parameters.

All study participants were informed about the nature of the study, and their oral and written consents to participating voluntarily were obtained. The study was approved by the ethical committee of the Faculty of Medicine, Alexandria University. All study participants were subjected to a full demographic and medical history assessment with a detailed analysis of different cardiometabolic risk factors. Patients with incomplete or missing data were excluded and replaced with other eligible participants to maintain the predetermined sample size and ensure data completeness. 

Anthropometric measurements

The measurements were assessed with the patients wearing light clothing without shoes, using stretch-resistant tape. The measuring tape was checked for accuracy against a rigid ruler before measurements. Patients were asked to stand upright with heels together, arms at sides, and eyes ­looking straight forward in alignment with the Frankfort plane. All the measurements were taken twice, and the average of the two measurements was used in the study.

Body Mass Index

BMI was calculated as the body weight in kilograms divided by the height in meters squared. Patients were classified as normal (18.5-24.9 kg/m^2^), overweight (25.0-29.9 kg/m^2^), class I obesity (30.0-34.9 kg/m^2^), class II obesity (35.0-39.9 kg/m^2^), and class III obesity (≥40.0 kg/m^2^) [12].

Waist Circumference

WC was measured at a midpoint between the lower margin of the last palpable rib and the top of the iliac crest [12]. WC in males was categorized into normal (<94 cm), increased (<102 cm), and markedly increased (≥102 cm). In females, WC was categorized into normal (<80 cm), increased (<88 cm), and markedly increased (≥88 cm) [12].

Waist-to-Hip Ratio

Hip circumference was measured at the widest level over the femoral trochanters. WHR was calculated as WC divided by hip circumference. WHR in males was categorized into low risk (<0.90) and substantially increased risk (≥0.90). In females, WHR was categorized into low risk (<0.85) and substantially increased risk (≥0.85) [13].

Neck Circumference

NC was measured in the middle of the neck, below the cricoid cartilage, and afterwards at the level of the mid-cervical spine. Given the absence of standardized, validated NC cutoffs, we relied on thresholds reported in a prior Egyptian study to guide classification within our analysis to ensure contextual relevance. NC in males was categorized into normal (≤38.25 cm), overweight (38.26-40.25 cm), and obesity (>40.25 cm). In females, NC was categorized into normal (≤36.25 cm), overweight (36.26-37.25 cm), and obesity (>37.25 cm) [14].

Blood sampling

It was done in the morning (8.00-10.00 am) after an overnight fast of 10 h for assessment of the following: fasting plasma glucose (FPG), glycated hemoglobin (HbA1c), total cholesterol (TC), low-density lipoprotein cholesterol (LDL-C), high-density lipoprotein cholesterol (HDL-C), triglycerides (TG), serum creatinine, estimated glomerular filtration rate (eGFR), high sensitivity C-reactive protein (hs-CRP), and plasma fibrinogen level. The first-morning spot urine sample was collected to assess the urinary albumin-to-creatinine ratio (uACR).

Coronary angiography

Coronary angiography was performed through a transfemoral approach. Hemodynamically significant stenosis was diagnosed with 50% or more stenosis in the left main coronary artery (LMCA) and 70% or more stenosis in all other arteries. Among CAD patients, GS was calculated as an indicator of the severity of coronary stenosis. To calculate GS, each coronary stenosis was given a severity score as shown in Table 1.

**Table 1:** Severity scores assigned to coronary artery lesions based on percentage of stenosis in the Gensini scoring system The table is created by the authors of the study.

Reduction of lumen	Severity score
<25%	1
26 - 50%	2
51 -75%	4
76 - 90%	8
91 - 99%	16
Total occlusion	32

Then, each lesion score is multiplied by a factor that takes into account the importance of the lesion's position in the coronary circulation (Table 2).

**Table 2:** Coronary artery segment importance factors used in the Gensini scoring system LMCA: left main coronary artery; LAD: left anterior descending artery; LCX: left circumflex artery; RCA: right coronary artery The table is created by the authors of the study.

Coronary artery segment	Importance factor
LMCA	X 5
The proximal segment of the LAD	X 2.5
The proximal segment of the LCX	X 2.5
The mid-segment of the LAD	X 1.5
The RCA	X 1
The distal segment of the LAD	X 1
The posterior descending artery	X 1
The obtuse marginal artery	X 1
The first diagonal artery	X 1
Other segments	X 0.5

Finally, the GS is calculated by summing the individual coronary segment scores [15]. The patients were categorized into three groups based on the GS: mild CAD: <11, moderate CAD: 11-38, severe CAD: >38, according to a previously published classification.

Anthropometric and angiographic assessments were performed by trained personnel blinded to the CAD status of participants. To minimize inter- and intra-observer variability, standardized protocols were followed, and repeated measurements were taken where applicable.

Statistical analysis of the data

Data were analyzed using IBM SPSS Statistics for Windows, Version 20 (Released 2011; IBM Corp., Armonk, New York, United States). Qualitative data were described using numbers and percentages. The Kolmogorov-Smirnov test was used to verify the normality of the distribution. Quantitative data were described by mean and standard deviation. The significance of the obtained results was assessed at the 5% level. For categorical variables, the chi-square test was used to compare different groups. The Monte Carlo correction was used for corrections of chi-square when more than 20% of the cells have an expected count of less than five. The Student t-test was used for normally distributed quantitative variables to compare between two studied groups. The Mann-Whitney test was used for non-normally distributed quantitative variables to compare between two studied groups. The relationship between NC and other parameters was assessed by correlation coefficient analysis (Pearson correlation coefficient). The relationship between GS and other parameters was assessed by correlation coefficient analysis (Spearman correlation coefficient). To adjust for multiple comparisons, the false discovery rate (FDR) correction was applied using the Benjamini-Hochberg procedure with a significance threshold of Q = 0.05. Assumptions of the regression model were assessed using the Shapiro-Wilk test for normality, the Breusch-Pagan test for homoscedasticity, and the variance inflation factor (VIF) for multicollinearity.

## Results

Our study included 54 males and 26 females in the group of T2DM patients with CAD (cases). The mean age of CAD patients was 60.85 ± 9.59 years, and the mean DM duration was 12.78 ± 6.69 years. On the other hand, there were 52 males and 28 females in the group of T2DM patients without CAD (controls). The mean age of patients without CAD was 59.13 ± 9.05 years, and the mean DM duration was 13.34 ± 6.84 years. There was no statistically significant difference between the groups regarding patients’ demographic characteristics (Table 3). This matching was applied during the study design phase to reduce confounding. Although DM duration appeared roughly symmetric (skewness = 0.046), the Shapiro-Wilk test showed non-normality (p<0.001), so the non-parametric Mann-Whitney U test was used to compare DM duration between groups.

**Table 3:** Comparison between the two studied groups according to demographic characteristics and different cardiometabolic risk factors FDR: false discovery rate; HbA1c: glycated hemoglobin; LDL-C: low density lipoprotein cholesterol; HDL-C: high density lipoprotein cholesterol; eGFR: estimated glomerular filtration rate; uACR: urinary albumin to creatinine ratio; hs-CRP: high-sensitivity C-reactive protein; mg: milligrams; dl: deciliter; g: gram; ml: milliliters; min: minutes; m^2^: square-meter; g.Cr: gram creatinine; n: number; %: percentage; t: Student t-test; x2: chi-square test; U: Mann-Whitney test; p: p-value for comparing between the two studied groups; *: statistically significant at p ≤ 0.05, p-values were corrected for multiple comparisons using the FDR method. Bolded p-values remain significant after FDR correction.

Parameters	Categories	T2DM patients with CAD (n = 80)	T2DM patients without CAD (n = 80)	Test of significance	p-value	FDR-adjusted p-value
Sex						
	Male	54 (67.5%)	52 (65%)	χ^2 ^= 0.112	0.738	
	Female	26 (32.5%)	28 (35%)			
Age (years)		60.85 ± 9.59	59.13 ± 9.05	t = 1.170	0.244	
DM duration (years)		12.78 ± 6.69	13.34 ± 6.84	U = 3069.50	0.656	
Fasting plasma glucose (mg/dl)		177.1 ± 76.48	147.5 ± 50.21	t = 2.891*	0.004*	0.0073*
HbA1c (%)		8.54 ± 1.87	7.84 ± 1.45	t = 2.640*	0.009*	0.0141*
Total cholesterol (mg/dl)		163.4 ± 49.75	178.3 ± 48.73	t = 1.917	0.057	0.0697
Triglycerides (mg/dl)		154.3 ± 95.04	125.0 ± 47.73	t = 2.460*	0.015*	0.0206*
LDL-C (mg/dl)		92.21 ± 39.21	102.4 ± 40.27	t = 1.625	0.106	0.1166
HDL-C (mg/dl)		39.91 ± 9.33	41.36 ± 7.75	t = 1.069	0.287	0.2870
Creatinine (mg/dl)		1.0 ± 0.13	0.94 ± 0.10	t = 3.299*	0.001*	0.0028*
eGFR (ml/min/1.73 m^2^)		79.68 ± 9.80	86.33 ± 10.19	t = 4.208*	<0.001*	0.0028*
uACR (mg/g.Cr)		190.2 ± 364.5	57.26 ± 89.20	t = 3.170*	0.002*	0.0044*
hs-CRP (mg/dl)		10.21 ± 8.60	5.92 ± 5.17	t = 3.817*	<0.001*	0.0028*
Fibrinogen (mg/dl)		345.8 ± 93.58	300.1 ± 64.21	U = 2206.00*	0.001*	0.0028*

T2DM patients with CAD had a significantly higher mean FPG (p = 0.004), HbA1c (p = 0.009), TG (p = 0.015), serum creatinine (p = 0.001), uACR (p = 0.002), hs-CRP (p<0.001), and plasma fibrinogen (p = 0.001) and a significantly lower mean eGFR (p<0.001) compared to T2DM patients without CAD. There was no statistically significant difference between the groups regarding TC, LDL-C, and HDL-C levels. After applying FDR correction, FPG (adjusted p = 0.0073), HbA1c (adjusted p = 0.0141), TG (adjusted p = 0.0206), uACR (adjusted p = 0.0044), serum creatinine (adjusted p = 0.0028), eGFR (adjusted p = 0.0028), hs-CRP (adjusted p = 0.0028), and fibrinogen (adjusted p = 0.0028) remained statistically significant (FDR-adjusted p<0.05) (Table 3).

T2DM patients with CAD demonstrated significantly higher mean body weight (p = 0.049) and BMI (p = 0.013) compared to those without CAD. Additionally, WC was significantly greater in the CAD group (p<0.001), with a higher proportion of individuals exhibiting markedly increased WC (p = 0.039). Sex-specific analysis revealed that T2DM male patients with CAD had a significantly higher mean WC compared to males without CAD (p<0.001). In contrast, although female patients with CAD exhibited a higher mean WC than their non-CAD counterparts, the difference did not reach statistical significance (p = 0.316) (Table 4).

**Table 4:** Comparison between the two studied groups according to different anthropometric measurements FDR: false discovery rate; kg: kilogram, m^2^: square-meter; MC: Monte Carlo; cm: centimeter; n: number; %: percentage; x^2^: chi-square test; t: Student t-test; p: p value for comparing between the two studied groups; *: statistically significant at p ≤ 0.05, p-values were adjusted for multiple comparisons using the Benjamini-Hochberg FDR method. Bolded p-values remain significant after FDR correction.

Anthropometric measurement	Categories	T2DM patients with CAD (n = 80)	T2DM patients without CAD (n = 80)	Test of significance	p-value	FDR-adjusted p-value
Body weight (kg)		89.58 ± 11.24	86.10 ± 10.99	t = 1.977*	0.049*	0.049*
Body mass index (kg/m^2^)		30.88 ± 3.22	29.62 ± 3.10	t = 2.523*	0.013*	0.0217*
	Normal (18.5 - 24.9)	2 (2.5%)	6 (7.5%)	χ^2^ = 5.254	^MC^P= 0.148	
	Overweight (25 - 29.9)	27 (33.8%)	36 (45%)			
	Obese I (30 - 34.9)	41 (51.3%)	32 (40%)			
	Obese II (35 - 39.9)	10 (12.5%)	6 (7.5%)			
Waist circumference (cm)						
Males		109.0 ± 7.28	102.8 ± 8.22	t = 4.099^*^	<0.001^*^	
	Normal ( <94)	2 (3.7%)	7 (13.5%)	χ^2 ^= 5.845	^MC^p= 0.062	
	Increased (<102)	6 (11.1%)	11(21.2%)			
	Markedly increased (≥102)	46 (85.2%)	34 (65.4%)			
Females		102.3 ± 10.91	99.25 ± 11.51	t = 1.013	0.316	
	Normal (<80)	1 (3.8%)	2 (7.1 %)	χ^2 ^= 1.023	^MC^p= 0.749	
	Increased (<88)	2 (7.7 %)	4 (14.3%)			
	Markedly increased (≥88)	23 (88.5 %)	22 (78.6 %)			
Total		106.8 ± 9.10	101.6 ± 9.58	t = 3.561*	<0.001*	0.0025*
	Normal	3 (3.8%)	9 (11.3%)	χ^2^ = 6.482*	0.039*	
	Increased	8 (10%)	15 (18.8%)			
	Markedly increased	69 (86.3%)	56 (70%)			
Waist-to-hip ratio						
Males		0.93 ± 0.04	0.91 ± 0.04	t = 2.256^*^	0.026^*^	
	Low risk (<0.9)	13 (24%)	20 (38.5%)	χ^2 ^= 2.5576	0.109	
	High risk (≥0.9)	41 (76%)	32 (61.5%)			
Females		0.85 ± 0.05	0.82 ± 0.05	t = 1.695	0.096	
	Low risk (<0.85)	6 (23%)	10 (35.7%)	χ^2 ^= 1.0326	^MC^p= 0.309	
	High risk (≥0.85)	20 (77%)	18 (64.3%)			
Total		0.90 ± 0.06	0.88 ± 0.06	t = 2.326^*^	0.021^*^	0.0263*
	Low risk	19 (23.75%)	30 (37.5%)	χ^2 ^= 3.559	0.059	
	High risk	61 (76.25%)	50 (62.5%)			
Neck circumference (cm)						
Males		42.61 ± 2.95	40.71 ± 2.78	t = 3.411^*^	0.001^*^	
	Normal (≤38.25)	4 (7.4%)	9 (17.3%)	χ^2 ^= 10.956^*^	0.004^*^	
	Overweight (>38.25)	11 (20.4%)	22 (42.3%)			
	Obesity (>40.25)	39 (72.2%)	21 (40.4%)			
Females		39.08 ± 2.72	37.95 ± 2.51	t = 1.587	0.118	
	Normal (≤36.25)	2 (7.7%)	6 (21.4%)	χ^2 ^= 3.981	^MC^p= 0.144	
	Overweight (>36.25)	6 (23.1%)	10 (35.7%)			
	Obesity (>37.25)	18 (69.2%)	12 (42.9%)			
Total		41.46 ± 3.31	39.74 ± 2.98	t = 3.450^*^	0.001^*^	0.0025*
	Normal	6 (7.5%)	15 (18.8%)	χ^2 ^= 14.849^*^	0.001^*^	
	Overweight	17 (21.3%)	32 (40%)			
	Obesity	57 (71.3%)	33 (41.3%)			

In comparison to T2DM patients without CAD, T2DM patients with CAD had a significantly higher mean WHR (p = 0.021). When stratified by sex, male T2DM patients with CAD had a significantly greater mean WHR than males without CAD (p = 0.026). Among females, although those with CAD had a higher mean WHR compared to those without CAD, the difference was not statistically significant (p = 0.096) (Table 4).

T2DM patients with CAD demonstrated a significantly higher mean NC (p = 0.001) and a significantly greater proportion of individuals classified as obese based on NC cutoff values (p = 0.001), compared to those without CAD (Table 4). When stratified by sex, male T2DM patients with CAD had a significantly higher mean NC (p = 0.001) and a much larger percentage of patients with obesity as determined by NC cutoff (p = 0.004), whereas in females, the higher mean NC observed in CAD patients did not reach statistical significance (p = 0.118) (Table 4).

After applying FDR correction, body weight (adjusted p = 0.049), BMI (adjusted p = 0.0217), WC (adjusted p = 0.0025), WHR (adjusted p = 0.026), and NC (adjusted p = 0.0025) remained statistically significant (FDR-adjusted p<0.05) (Table 4).

NC showed a strong positive correlation with other anthropometric measurements such as body weight (r = 0.636, p<0.001), WC (r = 0.578, p<0.001), and WHR (r = 0.675, p<0.001) and a moderate positive correlation with BMI (r = 0.482, p<0.001). Also, NC showed a weak positive correlation with both FPG (r = 0.162, p = 0.040) and HbA1c (r = 0.285, p<0.001). The correlation with FPG was not significant after FDR correction (r = 0.162, adjusted p = 0.09333), while other parameters, including body weight (adjusted p = 0.00028), BMI (adjusted p = 0.00028), WC (adjusted p = 0.00028), WHR (adjusted p = 0.00028), HbA1c (adjusted p = 0.00028) remained statistically significant. No significant correlations were found with lipid profile, eGFR, uACR, hs-CRP, or fibrinogen (Table 5).

**Table 5:** Correlation between neck circumference and different studied parameters FDR: false discovery rate; HbA1c: glycated hemoglobin; LDL-C: low density lipoprotein cholesterol; HDL-C: high density lipoprotein cholesterol; eGFR: estimated glomerular filtration rate; uACR: urinary albumin to creatinine ratio; hs-CRP: high-sensitivity C-reactive protein; kg: kilogram; m^2^: square meter; mg: milligrams; dl: deciliter; ml: milliliters; g.Cr: gram creatinine; cm: centimeter, min: minutes; r: Pearson coefficient; p: p-value for comparing the two studied groups; *: statistically significant at p ≤ 0.05, p-values were corrected for multiple comparisons using the Benjamini-Hochberg FDR method. Bolded values remained statistically significant at FDR-adjusted p<0.05.

Parameters	r	p	FDR-adjusted p-value
Body weight (kg)	0.636*	<0.001*	0.00028*
Body mass index (kg/m^2^)	0.482*	<0.001*	0.00028*
Waist circumference (cm)	0.578*	<0.001*	0.00028*
Waist-to-hip ratio	0.675*	<0.001*	0.00028*
Fasting plasma glucose (mg/dl)	0.162*	0.040*	0.09333
HbA1c (%)	0.285*	<0.001*	0.00028*
Total cholesterol (mg/dl)	-0.051	0.518	0.60433
Triglycerides (mg/dl)	0.055	0.491	0.60433
LDL-C (mg/dl)	0.040	0.613	0.66015
HDL-C (mg/dl)	-0.109	0.172	0.26756
eGFR (ml/min/1.73 m^2^)	-0.075	0.346	0.48440
uACR (mg/g.Cr)	0.128	0.106	0.21200
hs-CRP (mg/dl)	0.111	0.160	0.26756
Fibrinogen (mg/dl)	-0.011	0.892	0.89200

The mean GS in T2DM patients with CAD was 41.89 ± 32.76. Patients were further classified according to GS into: patients with mild CAD (GS<11) who represented 22.5% of the total patients, patients with moderate CAD (GS 11-38) who represented 37.5% of the total patients and patients with severe CAD (GS>38) who represented 40% of the total patients (Table 6).

**Table 6:** Descriptive analysis of T2DM patients with CAD (group A) according to GS n: number; %: percentage; min: minimum; max: maximum; SD: standard deviation; IQR: interquartile range; CAD: coronary artery disease; T2DM: type 2 diabetes mellitus; GS: Gensini score

Gensini score	No.	%
Mild CAD (<11)	18	22.5
Moderate CAD (11 – 38)	30	37.5
Severe CAD (>38)	32	40.0
Min. – Max.	6.0 – 126.0	
Mean ± SD.	41.89 ± 32.76	
Median (IQR)	36.0 (13.0 – 64.0)	

GS showed a strong positive correlation with HbA1c (r = 0.545, p<0.001), and it weakly positively correlated with DM duration (r = 0.234, p = 0.037), FPG (r = 0.371, p = 0.001), and hs-CRP (r = 328, p = 0.003). NC was the only anthropometric measure that strongly positively correlated with GS (r = 0.685, p<0.001). The correlation with DM duration was not statistically significant after FDR correction (adjusted p = 0.1184), while other parameters, including NC (adjusted p = 0.0008), FPG (adjusted p = 0.0053), HbA1C (adjusted p = 0.0008), and hs-CRP (adjusted p = 0.0120), remained statistically significant. No significant correlations were observed with other studied parameters (Table 7).

**Table 7:** Correlation between Gensini score and different studied parameters FDR: false discovery rate; HbA1c: glycated hemoglobin; LDL-C: low density lipoprotein cholesterol; HDL-C: high density lipoprotein cholesterol; eGFR: estimated glomerular filtration rate; uACR: urinary albumin to creatinine ratio; hs-CRP: high-sensitivity C-reactive protein; kg: kilogram; mg: milligrams; dl: deciliter; ml: milliliters; min: minutes; m^2^: square-meter; g.Cr: gram creatinine; cm: centimeter; r_s_: Spearman coefficient, p: p-value for comparing the two studied groups, *: statistically significant at p ≤ 0.05, p-values were corrected for multiple comparisons using the Benjamini-Hochberg FDR method. Bolded values remained statistically significant at FDR-adjusted p<0.05.

Parameters	r_s_	p	FDR-adjusted p-value
DM duration (years)	0.234	0.037*	0.1184
Body weight (kg)	0.120	0.289	0.6606
Body mass index (kg/m^2^)	-0.027	0.813	0.9680
Waist circumference (cm)	0.015	0.897	0.9680
Waist-to-hip ratio	0.203	0.071	0.1893
Neck circumference (cm)	0.685	<0.001*	0.0008*
Fasting plasma glucose (mg/dl)	0.371	0.001^*^	0.0053*
HbA1c (%)	0.545	<0.001*	0.0008*
Total cholesterol (mg/dl)	0.009	0.936	0.9680
LDL-C (mg/dl)	0.109	0.337	0.6740
HDL-C (mg/dl)	0.063	0.579	0.8422
Triglycerides (mg/dl)	-0.078	0.494	0.7904
eGFR (ml/min/1.73 m^2^)	-0.099	0.382	0.6791
uACR (mg/g.Cr)	0.005	0.968	0.9680
hs-CRP (mg/dl)	0.328	0.003*	0.0120*
Fibrinogen (mg/dl)	0.019	0.870	0.9680

Given the non-normal distribution and potential non-linearity in the relationship between GS and different studied parameters, Spearman’s correlation was used as a robust measure of association.

The univariate analysis of the different factors affecting GS showed that DM duration (p = 0.049), FPG (p<0.001), HbA1c (p<0.001), hs-CRP (p = 0.036), and NC (p<0.001) were each independently significantly and positively correlated with GS. Multivariate regression analysis of these factors revealed, after adjusting for confounding variables including age, sex, socioeconomic status and smoking status, that DM duration (p = 0.046), FPG (p<0.001), hs-CRP (p = 0.002), and NC (p<0.001) remained independently associated with GS (Table 8).

**Table 8:** Univariate and multivariate analysis for the parameters affecting the Gensini score B: unstandardized coefficients; C.I: confidence interval; #: all variables with p<0.05 were included in the multivariate; p: p-value, *: statistically significant at p ≤ 0.05; HbA1c: glycated hemoglobin; %: percentage; LDL-C: low density lipoprotein cholesterol; HDL-C: high density lipoprotein cholesterol; eGFR: estimated glomerular filtration rate; uACR: urinary albumin to creatinine ratio; hs-CRP: high-sensitivity C-reactive protein; kg: kilogram; mg: milligrams; dl: deciliter; ml: milliliters; min: minutes; m^2^: square-meter; g.Cr: gram creatinine; cm: centimeter

Parameters	Univariate		#Multivariate	
	p	B (95%C.I)	p	B (95% CI)
DM duration (years)	0.049^*^	1.081 (0.004 – 2.157)	0.046^*^	0.668 (0.013 – 1.323)
Body weight (kg)	0.256	0.374 (-0.277 – 1.026)	-	-
Body mass index (kg/m^2^)	0.848	0.221 (-2.070 – 2.512)	-	-
Waist circumference (cm)	0.630	0.197 (-0.613 – 1.007)	-	-
Waist-to-hip ratio	0.083	112.846 (-15.163 – 240.854)	-	-
Neck circumference (cm)	<0.001^*^	6.869 (5.262 – 8.477)	<0.001^*^	5.318 (3.894 – 6.742)
Fasting plasma glucose (mg/dl)	<0.001^*^	0.228 (0.147 – 0.310)	<0.001^*^	0.138 (0.070 – 0.205)
HbA1c (%)	<0.001^*^	9.552 (6.242 – 12.862)	0.214	1.846 (-1.087 – 4.778)
Total cholesterol (mg/dl)	0.776	0.021 (-0.127 – 0.170)	-	-
LDL-C (mg/dl)	0.523	0.061 (-0.127 – 0.248)	-	-
HDL-C (mg/dl)	0.350	0.372 (-0.416 – 1.159)	-	-
Triglycerides (mg/dl)	0.311	-0.040 (-0.117 – 0.038)	-	-
eGFR (ml/min/1.73 m^2^)	0.436	-0.295 (-1.046 – 0.456)	-	-
uACR (mg/g.Cr)	0.609	0.005 (-0.015 – 0.025)	-	-
hs-CRP (mg/dl)	0.036^*^	0.895 (0.060 – 1.730)	0.002^*^	0.824 (0.315 – 1.332)
Fibrinogen (mg/dl)	0.848	-0.008 (-0.087 – 0.071)	-	-

Although HbA1c was statistically significant in the univariate model, it lost significance in the multivariate model (p = 0.214), possibly due to collinearity with FPG. While VIF did not indicate problematic multicollinearity overall, the overlap between glycemic indicators (HbA1c and FPG) may have reduced the independent contribution of HbA1c to the model.

Assumption diagnostics for the multiple linear regression model were conducted to ensure the validity of the results. The Shapiro-Wilk test indicated a statistically significant deviation from normality of residuals (p = 0.001); however, visual inspection suggested approximate normal distribution. The Breusch-Pagan test showed no evidence of heteroscedasticity (p = 0.80). Multicollinearity was assessed using VIF, with all values within acceptable limits, indicating no significant multicollinearity among the predictors. Additionally, Cook’s distance values did not suggest the presence of influential outliers. The model demonstrated good explanatory power, with an R² of 0.745 and an adjusted R² of 0.680.

NC was independently associated with GS, with a regression coefficient of B = 5.318, indicating that each 1 cm increase in NC was associated with an average increase of 5.318 points in the GS. This effect size was larger than that of other included predictors, suggesting a potentially meaningful clinical relationship.

## Discussion

CAD is a serious public health problem and is one of the principal causes of death worldwide [1]. It is reasonable to adjust different modifiable CAD risk factors to reduce the ensuing financial and illness-related disease burden. Chronic hyperglycemia is strongly linked to higher adverse cardiovascular outcomes; thus, achieving better glycemic control might improve the clinical outcomes for patients with diabetes [16]. In our study, CAD patients exhibited poorer glycemic control. On reviewing the literature, several studies confirmed the tight association between poor glycemic control and CAD [16]. Chen et al. demonstrated that poor glycemic control in T2DM patients had a detrimental impact on the endothelial function and was associated with higher CAD risk [17].

Dyslipidemia is a very prevalent problem in T2DM patients, which also plays a significant role in CAD. Our study demonstrated an association between elevated TG levels and CAD (adjusted p = 0.0206), but there was no significant difference in TC, LDL-C, and HDL-C levels between T2DM patients with and without CAD. In agreement with our results, Tseng et al. concluded that elevated TG was the only lipid parameter that associated with CAD [18]. The lack of association between TC, LDL-C, or HDL-C and CAD may be partially attributed to the widespread use of statins among our CAD patients. Additionally, the influence of residual confounding factors, such as dietary intake and genetic variability, cannot be ruled out. Furthermore, the limited sample size may have reduced the statistical power to detect subtle but clinically relevant differences.

Chronic kidney disease (CKD) is considered a CAD risk factor, as CKD patients are exposed to non-traditional cardiovascular risk factors such as oxidative stress and inflammation [19]. Our study showed that CAD patients had a significantly higher serum creatinine (adjusted p = 0.0028) and lower eGFR (adjusted p = 0.0028). Numerous former studies had revealed a significant association between impaired kidney function and cardiovascular disease (CVD) incidence and severity [20]. Also, albuminuria is a confirmed CKD biomarker and is additionally documented as a CAD risk factor nowadays. Our study agreed with the literature and demonstrated that CAD patients had higher albuminuria (adjusted p = 0.0044). Also, Lin et al. demonstrated that cardiovascular mortality risk can be increased by even mildly increased uACR in T2DM patients [21].

It is becoming clear that hs-CRP contributes significantly to the pathogenesis of atherosclerosis and is a significant cardiovascular risk factor [22]. Additionally, fibrinogen is an emerging CAD risk factor, and different studies demonstrated the association between elevated hs-CRP and/or fibrinogen levels and CAD [22,23]. Growing evidence supports hs-CRP as a valuable marker of cardiovascular risk, as it is linked to the pathophysiology of atherosclerosis. A systematic review and meta-analysis, including a total of 20,395 CAD patients, found that higher fibrinogen levels are significantly linked to an increased risk of both cardiovascular and all-cause mortality in this population [23]. Our study also showed that both higher hs-CRP (adjusted p = 0.0028) and plasma fibrinogen (adjusted p = 0.0028) levels were significantly associated with CAD in T2DM patients.

Obesity is considered an independent CAD risk factor as it can accelerate atherosclerotic processes through its impact on other major risk factors such as DM and dyslipidemia [2]. BMI is the most common obesity screening parameter, as it is an easy, affordable, reasonably accurate, and noninvasive tool. But BMI is not an accurate measure of body fat, as it assesses increased body weight rather than increased body fat, and it does not take into consideration body fat distribution, which is a key component of obesity-related disorders. Our study and several previous studies identified the significant impact of higher body weight and higher BMI on the incidence and severity of CAD [24].

In our study, although the difference in body weight between groups was modest (~3.5 kg), it reached statistical significance. Even small weight reductions can yield meaningful health benefits, especially when accompanied by variations in fat distribution, which may have a more direct impact on cardiovascular risk. For instance, losing as little as 1 kg has been associated with improvements in glycemic control, insulin sensitivity, and cardiovascular risk factors in patients with T2DM [25].

Both WC and WHR are considered surrogate measures of abdominal fat mass and are associated with a higher incidence of several cardiometabolic diseases [10]. Our study's findings supported previous research by showing that CAD patients had considerably greater WC (adjusted p = 0.0025) and WHR (adjusted p = 0.0263). Harwalkar et al. stated that the association between anthropometric measures such as BMI, WC, and WHR and CAD suggests they are strong predictors of disease onset. Therefore, these simple indicators can be effectively used to identify individuals at risk and guide timely preventive interventions [26]. Regarding the sex difference in the association between CAD and WC, Cameron et al. agreed with our results and concluded, in a prospective study of 6,072 subjects, that increased WC was associated with a higher risk of CAD in men but not in women when adjusted for BMI and other covariates [27]. The lack of a significant association between WC or WHR and CAD in females may be attributed to the limited number of female participants in our study, which may have reduced the statistical power needed to detect meaningful associations. The major drawback of WC and WHR is that they are affected by different causes of abdominal distension, such as gaseous distension. Respiratory movement and tight clothing can also hinder the precise measurements of WC.

NC is a novel anthropometric parameter representative of upper-body adiposity and is also a powerful marker of abdominal obesity [4]. NC is a more practical and better indicator for assessing obesity than BMI and WC, as it overcomes many of the drawbacks of the two techniques. Our study further showed evidence of the usefulness of NC as a screening tool for overweight and obesity, as it revealed a substantial correlation between NC and other obesity indices such as body weight (adjusted p = 0.0028), BMI (adjusted p = 0.0028), WC (adjusted p = 0.0028), and WHR (adjusted p = 0.0028). In line with our results, an Egyptian cross-sectional study, including 6718 adult Egyptian subjects, showed a strong correlation between NC and body weight, height, WC, hip circumferences, BMI, and WHR [13]. 

The relationship between NC and CAD has been thoroughly investigated over the past years. Recently, it has been increasingly recognized that NC is independently linked to several metabolic risk factors and metabolic-related comorbidities and that it is substantially correlated with CVD [28]. In the present study, we found that T2DM patients with CAD had significantly higher NC measurements (adjusted p = 0.0025), and these results are in concordance with most of the previous studies. Yang et al. demonstrated, in a prospective multi-center study including 3299 T2DM patients, that larger NC was associated with CVD both at baseline and during 10-year management and that larger NC can increase CAD by about 40% in T2DM [8]. Similarly, a meta-analysis that included eight observational studies demonstrated that larger NC was associated with a higher risk of CAD, particularly when coronary angiography was used to diagnose CAD [29]. Regarding the sex difference in the association between CAD and NC, Hu et al. agreed with our results and demonstrated that there was a significant association between NC and cardiovascular events in men but not in women [30]. The absence of a significant association between NC and CAD in females could be due to the small number of female participants in our study, potentially limiting the statistical power to identify meaningful relationships. Additionally, sex-specific differences in fat distribution and hormonal influences may contribute to variability in how NC reflects cardiovascular risk among women, potentially affecting the strength of the observed relationship.

On analyzing the association between NC and different cardiometabolic risk factors, we found that NC was positively correlated with parameters of glycemic control, such as HbA1c (adjusted p = 0.0028). In line with our results, a meta-analysis, including 21 studies, proved the significant correlation between NC and HbA1c [31].

GS is an effective tool used to evaluate the severity of CAD. The GS was selected for its ability to quantify the severity of CAD by accounting for both the degree of luminal narrowing and the anatomical importance of lesion location. Compared to other scoring systems, such as SYNTAX, which is primarily used for guiding revascularization strategy, the GS offers a more comprehensive assessment of atherosclerotic burden, making it more suitable for evaluating disease severity in observational studies. Our study showed that there was a significant positive correlation between GS and FPG (adjusted p = 0.0053), HbA1c (adjusted p = 0.0008), and hs-CRP (adjusted p = 0.0120). NC was the only anthropometric measure that had a significant correlation with GS (adjusted p = 0.0008). The relationship between GS and glycemic control is well established, and most of the previous studies confirmed the association between GS and HbA1c even in non-diabetics [16]. Kamal et al. proved that GS was positively correlated with FBG and HbA1C [32]. Regarding the relation between GS and hs-CRP, our study results were consistent with previous research findings. Peppes et al. demonstrated that there were significant positive correlations between inflammatory biomarkers, including hs-CRP and GS [33].

After an extensive literature search, we found that there were no studies on the association between NC and CAD severity as assessed by GS. However, a number of other studies that used scores other than GS to evaluate the relationship between NC and CAD severity were carried out. Kaulgud et al. proved that there was a significant correlation between CAD severity as assessed by Jenkins’ scores and NC [34]. Çınaroğlu et al. concluded that CAD severity, as determined by the SYNTAX score, was correlated with the NC [35]. Tian et al. proved that increasing NC quartiles were significantly associated with the severity of CAD as assessed by the number of diseased coronary vessels [36].

NC was the sole anthropometric parameter that demonstrated a significant association with the GS. It is likely because NC reflects both VAT and upper-body SAT, both of which are metabolically active and strongly linked to CVD. This dual association may explain its stronger predictive value for CAD severity compared to other anthropometric measures. Also, NC is not affected by factors like abdominal distension, which may make it a more stable and reliable measure compared to other anthropometric indices. The observed effect of NC on CAD severity, as reflected by a regression coefficient of 5.318, highlights its potential clinical relevance. However, as the study is observational and based on a case-control design, causality cannot be established. These findings represent associations only, and further longitudinal or interventional studies are required to validate and expand upon these results.

Our study has some limitations. The first limitation is the small sample size, especially among female participants. Therefore, future studies with larger and more balanced samples will be necessary to validate and expand upon our findings. The second limitation is that the study was conducted exclusively on Egyptian patients attending the catheter lab of Alexandria Main University Hospital. As such, the findings may not be fully generalizable to populations of different ethnic or geographic backgrounds. Further research incorporating ethnically and geographically diverse populations is warranted to confirm and generalize these findings. The third limitation is that the power calculation was based on prevalence for group comparison, which may not fully reflect the power needed for analyzing continuous outcomes like the correlation between NC and GS. The fourth limitation is that the study did not account for potential confounding factors such as physical activity, dietary habits, and medication use, which may influence the relationship between anthropometric measures and CAD severity. Future studies should aim to include and control for these variables to enhance the validity of the findings.

## Conclusions

NC is considered a promising tool to assess obesity as it is simple, reliable, easy to measure, and a widely available anthropometric measure. NC is a very economical method, so it can be applied in screening programs for obesity in remote areas where the means of measuring weight and height are unavailable. NC showed a strong correlation with the severity of CAD, suggesting that it could be used as a risk assessment tool. Measuring NC may be considered a complementary procedure in clinical practice. Although the findings are encouraging, the single-center design and small sample size highlight the need for larger, multi-center studies to confirm the clinical value of NC.
